# Combining Prospective Acquisition CorrEction (PACE) with retrospective correction to reduce motion artifacts in resting state fMRI data

**DOI:** 10.1002/brb3.1341

**Published:** 2019-07-11

**Authors:** Pradyumna Lanka, Gopikrishna Deshpande

**Affiliations:** ^1^ Department of Electrical and Computer Engineering AU MRI Research Center, Auburn University Auburn Alabama; ^2^ Department of Psychological Sciences University of California Merced California; ^3^ Department of Psychology Auburn University Auburn Alabama; ^4^ Center for Health Ecology and Equity Research Auburn University Auburn Alabama; ^5^ Alabama Advanced Imaging Consortium, Auburn University and University of Alabama Birmingham Alabama; ^6^ Center for Neuroscience Auburn University Auburn Alabama; ^7^ Department of Psychiatry National Institute of Mental Health and Neurosciences Bangalore India

**Keywords:** functional connectivity, head motion, motion artifacts, prospective motion correction, resting state fMRI

## Abstract

**Background:**

Head movement in the scanner causes spurious signal changes in the blood‐oxygen‐level‐dependent (BOLD) signal, confounding resting state functional connectivity (RSFC) estimates obtained from functional magnetic resonance imaging (fMRI). We examined the effectiveness of Prospective Acquisition CorrEction (PACE) in reducing motion artifacts in BOLD data.

**Methods:**

Using PACE‐corrected RS‐fMRI data obtained from 44 subjects and subdividing them into low‐ and high‐motion cohorts, we investigated voxel‐wise motion‐BOLD relationships, the distance‐dependent functional connectivity artifact and the correlation between head motion and connectivity metrics such as posterior cingulate seed‐based connectivity and network degree centrality.

**Results:**

Our results indicate that, when PACE is used in combination with standard retrospective motion correction strategies, it provides two principal advantages over conventional echo‐planar imaging (EPI) RS‐fMRI data: (a) PACE was effective in eliminating significant negative motion‐BOLD relationships, shown to be associated with signal dropouts caused by head motion, and (b) Censoring with a lower threshold (framewise displacement >0.5 mm) and a smaller window around the motion corrupted time point provided qualitatively equivalent reductions in the motion artifact with PACE when compared to a more conservative threshold of 0.2 mm required with conventional EPI data.

**Conclusions:**

PACE when used in conjunction with retrospective motion correction methods including nuisance signal and motion parameter regression, and censoring, did prove effective in almost eliminating head motion artifacts, even with a lower censoring threshold. Use of a lower censoring threshold could provide substantial savings in data that would otherwise be lost to censoring. Three‐dimensional PACE has negligible overhead in terms of scan time, sequence modifications or additional and hence presents an attractive option for head motion correction in high‐throughput resting‐state BOLD imaging.

## 1 INTRODUCTION

Head motion is one of the major sources of artifacts in functional magnetic resonance imaging (fMRI). Head motion is said to cause large spatially varying signal changes across the brain. Realignment corrects the changes in brain position, but it does not take into consideration the changes in the image intensity associated with motion. Head motion, particularly in the direction perpendicular to the slice selection is susceptible to artifacts due to magnetic field inhomogeneity and spin‐excitation history effects (Friston, Williams, Howard, Frackowiak, & Turner, 1996).

Resting state functional connectivity (RSFC) measures the synchronicity of the brain activity in different regions of the brain and has become quite popular in the last decade due to its sensitivity to development, aging, and pathology. However, motion can severely affect the validity of resting‐state fMRI (rs‐fMRI) studies, particularly in hyperkinetic populations with large head movements such as children, diseased, and the elderly (Satterthwaite et al., 2013; Yan et al., 2013). The motion‐induced variance changes in the blood‐oxygen‐level‐dependent (BOLD) signal could potentially drive RSFC metrics in the same direction as one would expect due to disease or aging, thus confounding its effects.

Most motion correction approaches are typically classified into prospective motion correction and retrospective motion correction. In prospective motion correction, the motion is corrected for before or during the acquisition of the volumes, whereas retrospective motion correction methods correct for motion after the acquisition of the volumes. Rigid‐body realignment, nuisance signal regression, modeling the effects of the head motion on the BOLD signal using motion parameters and removing the fitted response, temporal band‐pass filtering, motion censoring or spike regression, group‐level correction or some combinations of the above approaches are routinely used with varying degrees of success in retrospective motion correction (Power et al., 2014; Satterthwaite et al., 2013; Yan et al., 2013).

Realignment is the first step in retrospective motion correction. Though it aims to make each voxel correspond to the same region in the brain in the fMRI time series by selecting a suitable rigid‐body transformation, it does not eliminate the changes in the image intensity associated with head motion. In nuisance signal regression, we regress out the mean signal corresponding to areas of cerebrospinal fluid (CSF), white matter (WM), and the global signal (GS) from the BOLD signal, with the hope that we would remove the variance of nonneural origin attributable to head motion, heartbeat, respiration, and other sources of scanner noise. Unlike regression of WM and CSF, global signal regression (GSR) has been reported in previous studies to remove significant amount of variance associated with head motion from the BOLD signal (Power et al., 2014; Satterthwaite et al., 2013; Yan et al., 2013). However, there have been some concerns that GSR introduces an artificial negative bias in the correlation coefficients, driving them downward everywhere in the brain to the point of introducing spurious anticorrelations (Murphy, Birn, Handwerker, Jones, & Bandettini, 2009) and is also said to create artifactual group differences in functional connectivity (Saad et al., 2012).

Spin history effects result in the signal intensity of the current acquisition to become a complex nonlinear function of the current position, as well as previous positions (Friston et al., 1996). In order to address this, one could model the effects of head movement on the BOLD signal by using a second order polynomial containing the current motion parameters and few previous (in time) motion parameters and remove the fitted response from the BOLD signal. Twenty‐four parameters (*R_t_*, $R_{t}^{2}$, *R_t‐1_*, $R_{t-1}^{2}$) and 36 parameters (*R_t_*, $R_{t}^{2}$, *R_t‐1_*, $R_{t-1}^{2}$, *R_t‐2_*, $R_{t-2}^{2}$) models, where *R* indicates the six realignment parameters, and *t* and the corresponding subscript indicates the number of volumes back in time used to correct for motion. The justification for using complex models is that it increases the fit compared to models using a lower number of parameters (Satterthwaite et al., 2013). However, going further back in time results in loss of degrees of freedom and may sometimes not justify the increase in fit. Also, fewer number of parameters might be appropriate for low‐motion subjects as increasing the number of parameters causes the model to over‐fit the data and thereby reduce the sensitivity to the underlying neural activity (Satterthwaite et al., 2013).

Finally, in motion censoring or scrubbing, the motion corrupted time points and the adjacent time points that exceed a threshold defined by a quality control metric derived from the data such as derivative of root mean squared variance over voxels (DVARS) or framewise displacement (FD, Power, Barnes, Snyder, Schlaggar, & Petersen, 2012; Power et al., 2014; Power, Schlaggar, & Petersen, 2012) are marked. Either the data for those time points are removed, or they are interpolated from the adjacent time points and removed after preprocessing. Similar to scrubbing, spike regression models the motion‐induced spikes in fMRI data and removes the fitted response, effectively eliminating the influence of the corrupted time points in the fMRI time series (Lemieux, Salek‐Haddadi, Lund, Laufs, & Carmichael, 2007; Satterthwaite et al., 2013). Scrubbing/censoring/spike regression, especially in high‐motion datasets could potentially lead to loss of a large quantity of data (Zeng et al., 2014) and in turn result in noisy estimates of the functional connectivity (Fair et al., 2013). Furthermore, scrubbing could introduce discontinuities in the data, which may invalidate many analysis methods used thereafter. Even though interpolation has been suggested as a way to avoid these discontinuities, interpolation is at best an approximation and still amounts to loss of original data. A summary of currently available retrospective motion correction methods and their effectiveness can be found in other papers (Goto et al., 2016; Power et al., 2012).

Given the difficulty in properly modeling the motion effects on the BOLD signal, prospective motion correction methods have gained increasing prominence. Although prospective motion correction has been in vogue for more than a decade, recent research (the flurry of articles that have appeared since Power et al., 2012) demonstrating the inadequacy of retrospective methods (most glaringly the rigid body realignment approach and motion parameter regression) suggests that it is imperative to evaluate prospective motion correction approaches in the context of motion effects on rs‐fMRI. Most prospective methods estimate the position of the head during scanning by using external‐tracking devices (Forman, Aksoy, Hornegger, & Bammer, 2011; Ooi, Krueger, Muraskin, Thomas, & Brown, 2011; Rotenberg et al., 2013; Todd, Josephs, Callaghan, Lutti, & Weiskopf, 2015; Zaitsev, Dold, Sakas, Hennig, & Speck, 2006). A review of prospective motion correction methods in fMRI can be found in Zaitsev, Akin, LeVan, & Knowles, 2017. Since these methods use an external device to independently record head motion and correct the gradients in near real‐time, they require elaborate setups, the subjects to wear a “marker” and sequence modification. Consequently, they are unsuitable for high‐throughput routine scanning. Alternatively, Prospective Acquisition CorrEction (PACE) is an image‐based online motion detection and correction sequence which tracks the subject's head location to keep the head position fixed relative to the scanner coordinate frame thereby reducing spin history effects associated with head motion (Thesen, Heid, Mueller, & Schad, 2000). Using an image‐based motion detection algorithm, the head motion parameters are estimated and fed back into the scanner so that the slice positioning and orientation are adjusted before the acquisition of the next volume. PACE accounts for motion based on the current volume realignment parameters and adjusts the position for the next volume acquisition by feeding back the calculated changes in head position to the measurement system. Since the position of the previous volume is used to acquire the current volume, there is a residual motion that cannot be accounted by PACE. That said, it requires no additional setup in terms of external devices, does not require subjects to wear any “targets” and is a functionality that is in‐built in FDA‐approved echo‐planar imaging (EPI) sequences on Siemens scanners (and hence does not require a sequence modification). For all these reasons, EPI‐PACE is suitable for high‐throughput routine imaging and therefore is worthy of being evaluated in the context of head motion artifacts in rs‐fMRI.

We had two main goals for this paper. First, we were particularly interested in understanding the effects of high and low head motion on RS‐fMRI data acquired using an EPI‐PACE sequence, using (a) the spurious motion‐BOLD relationships (Yan et al., 2013), (b) the motion‐induced distance‐dependent functional connectivity artifact (Power et al., 2012, 2014; Satterthwaite et al., 2012, 2013), and (c) the effect of motion on RS‐fMRI connectivity based metrics such as network degree centrality (DC) and posterior cingulate cortex seed‐based functional connectivity (PCC‐FC). Second, we examined if a combination of prospective and retrospective motion correction methods could do a better job of reducing motion artifacts in the BOLD data.

## 2 METHODS

### 2.1 Subjects

A total of 47 healthy adult subjects (20 males/27 females, age 25.1 ± 5 years) with no history of any neurological disorders were selected for this study. The subjects were instructed to relax, keep their eyes open, not think about anything in specific and keep their head as still as possible for the duration of the scans. Appropriate padding was provided to keep the head as still as possible in the scanner. All subjects gave informed consent, and the scanning procedure was performed in accordance with the guidelines and the approval of the Institutional Review Board at Auburn University.

### 2.2 Data acquisition

All subjects were scanned with a 3T MAGNETOM Verio scanner (Siemens Healthcare, Erlangen, Germany) using an EPI‐PACE sequence with a 32 channel head coil and the following acquisition parameters: TR of 1,000 ms, TE of 29 ms, Flip Angle of 90° with 16 slices, matrix = 64 × 64, voxel size = 3.5 × 3.5 × 5 mm^3^. The number of time points acquired for each subject ranged from 250 to 1,000. A T1 weighted MPRAGE anatomical image (TE = 2 ms, TR = 1900 ms, 176 slices with 1 × 1 × 1 mm^3^ voxel size) was also acquired for all the subjects to aid in spatial normalization.

### 2.3 Preprocessing of the RS‐fMRI data

The preprocessing of the RS‐ fMRI data was performed using Data Processing Assistant for Resting‐State fMRI (DPARSF) toolbox (Yan & Zang, 2010). The first five time points were removed from the time series to allow for T1 equilibration. Slice timing correction was applied to each slice in every volume to account for the different acquisition times of the slices. The volumes were then realigned using a six‐parameter (three translations, three rotations) rigid body transformation to account for the head motion by optimizing the minimum squared difference cost function by a two‐pass procedure. After realignment, the T1‐weighted anatomical image from each subject was registered to the mean functional image. Linear and quadratic detrending were performed to remove low‐frequency drift. Mean WM and CSF signals were regressed from the time series to remove non‐BOLD related signal variance. Also, the 24‐parameter motion regression proposed by Friston (Friston‐24) consisting of the six realignment parameters, their temporal derivatives and the squares of them, were regressed from the resting state fMRI BOLD time series. The preprocessed data are released publicly and can be obtained from Lanka & Deshpande, 2018.

### 2.4 Calculation of DVARS and head motion metrics

DVARS is the square root of mean square value of the temporal derivative of the intensities of the BOLD signal, calculated backward from the current time point to the previous time point over a voxel, ROI or the entire brain (Power et al., 2012, 2014). Traditionally, motion metrics are calculated from the realignment parameters, and their accuracy is limited by the accuracy of the estimates of the realignment parameters. Common metrics which capture subject head motion are the total displacement (TD) and FD. TD is measure of the change in the position of the head from its initial position, while FD is a measure of the change in the position of the head from the previous time point to the current time point and is calculated using realignment parameters of both the time points. It measures relative displacement rather than the absolute displacement of the head. In the case of motion correction by PACE, since the slice positioning is adjusted on the fly for every volume, these realignment parameters, and the FD metrics are a measure of the residual motion, relative to the scanner that is uncorrected by PACE rather than the actual motion of the head. Since all voxels in the brain do not move in a similar direction, it is essential to capture the individual movements of the voxel to understand the localized changes in signal intensities. So, along with volumetric measures the head's FD which assigns a single value of head motion to the entire brain, we also calculated the voxel‐specific framewise displacement (FD_vox_) which uses the six realignment parameters to estimate the relative displacement of every voxel at each time point. This enabled the computation of the displacement of each voxel with respect to the previous time point. More details on this approach are available in other papers (Satterthwaite et al., 2013; Yan et al., 2013). The following motion metrics were calculated for each subject for every time point: FD_FSL_ (Jenkinson, Bannister, Brady, & Smith, 2002) and FD_Power_ (Power et al., 2012), which are volume specific metrics. FD_Power_ and FD_FSL_ uses both translation and rotational parameters to estimate head motion. FD_Power_ assumes the radius of the head as 50 mm while FD_FSL_ assumes it to be 80 mm (Yan et al., 2013). We also estimated the spatial mean of FD_vox_ all voxels in the brain for every time point to calculate the voxel‐wise mean of framewise displacement (mean_sp_FD_vox_, Yan et al., 2013). Compared to FD_Power_, FD_FSL_ is more correlated with mean_sp_FD_vox_, indicating that it is a more appropriate summary statistic of the subject's head motion between two time points, though FD_Power_ has been used more often for marking and removing high‐motion time points from the data (Power et al., 2012, 2014). We also calculated the mean_sp_TD_vox_, which is the voxel‐wise mean of the TD of the brain in the scanner. Comparisons of different FD measures can be found in Yan et al., 2013.

### 2.5 Examination of motion‐BOLD relationships in PACE data

As voxel displacement is not spatially constant across the brain due to the combination of head rotations and head translations, voxel‐wise analysis of the motion‐BOLD relationships would be more appropriate to study the localized effect of head motion on BOLD signal intensity. Therefore, to understand the spatially varying relationships between head motion and the BOLD signal, the BOLD signal was preprocessed with several combinations of nuisance signal regressors: (a) CSF + WM regression, (b) CSF + WM + GS regression, (c) CSF + WM + Friston‐24 motion regression, (d) CSF + WM + GS + Friston‐24 motion regression, (d) CSF + WM + Friston‐24 motion regression + motion censoring (FD_Power_ threshold >0.5 mm and one back and two forward volumes regressed from the model), and (e) CSF + WM+GS + Friston‐24 motion regression + motion censoring. These pipelines were evaluated for each of the 44 subjects. Three of the subjects out of a the total 47 subjects, were eliminated because they did not have the necessary 3 min of data required for stable estimation of RSFC metrics after censoring (Yan et al., 2013). The Pearson's correlation coefficient was calculated between FD_vox_ and the BOLD signal for every voxel (preprocessed using the six pipelines mentioned above), and for all the volumes in the time series as described by Yan et al., 2013. With motion censoring, the same volumes which were removed from the BOLD signal were also removed from the FD_vox_ to calculate voxel‐wise correlation between motion and BOLD. Fischer's *z* transformation was performed on the resultant correlation maps to improve the normality of the data distribution. The resultant z‐maps were then normalized to the standard MNI template (3 mm^3^ cubic voxels) and the resulting volumes were smoothed with a 4.5 mm^3^ Gaussian kernel. A one‐sample *t* test was performed on the normalized correlation maps with a significance level of *p* < 0.05 (FDR corrected) to investigate consistent patterns of motion‐BOLD relationships within the group.

In order to further investigate the nature of the motion‐BOLD relationships for different levels of head motion, we divided our dataset into two subsets, a higher motion group (FD_FSL_ = 0.152 ± 0.062 mm) and a lower motion group (FD_FSL_ = 0.077 ± 0.014 mm) containing 22 subjects each based on their mean [FD_FSL_] with similar sex and age profiles in both the subgroups. We could have used any FD metric as they are highly correlated with each other, but used FD_FSL_ because of its relative convenience and accuracy (Yan et al., 2013). We then proceeded to repeat the procedure described above for both the datasets separately to understand the nature of the mostly artifactual motion‐BOLD signal relationships in the high‐motion and low‐motion datasets, with the expectation that motion‐BOLD relationships would be larger in magnitude and spatially distributed in the high‐motion group compared to the low‐motion group.

### 2.6 Examining the motion‐induced distance‐dependent artifact in functional connectivity

Head motion tends to distort functional connectivity metrics by inflating connectivity estimates between closer regions and reducing the connectivity between farther regions, as the voxels which are far from each other are less likely to experience similar movements, thus giving rise to the decaying effect of functional connectivity as a function of the head motion and the distance between them (Power et al., 2012; Satterthwaite et al., 2012). This is called as motion‐induced distance‐dependent artifact in functional connectivity throughout the paper. Power et al., (2012) reported that online motion correction by PACE did not ameliorate the distance‐dependent changes in functional connectivity induced by head motion. However, they did not show the corresponding results and did not elaborate it further. Therefore, to understand the effect of online motion correction on functional connectivity and to reveal the distance‐dependence artifact, we followed a procedure used previously (Satterthwaite et al., 2012, 2013) to characterize the effects of head motion artifacts in PACE data. After preprocessing the data, the volumes were normalized to the standard MNI template (3 mm^3^ cubic voxels) and were smoothed with a 4.5 mm^3^ Gaussian kernel, following which, the time series were filtered with a band‐pass filter with bandwidth of 0.01–0.1 Hz.

The 160 Regions of Interest (ROIs), as defined by the Dosenbach 160 atlas (Dosenbach et al., 2010), were extracted from the brain. Each ROI was modeled as a sphere with 10 mm diameter and the mean resting‐state preprocessed BOLD signal was obtained for each ROI. Functional connectivity was calculated as the correlation between the time series of every pair of ROIs, resulting in a functional connectivity matrix consisting of 12,720 elements ((160 × 160  – 160)/2) for each of the 44 subjects. These connectivity values were then correlated with the mean head motion obtained from each subject, that is, mean (FD_FSL_). These correlations were plotted on the y‐axis of a scatter plot with the Euclidean distance between ROIs on the x‐axis. The estimated correlation between FD and RSFC was then used to compare the success of PACE with a combination of retrospective motion correction methods to correct for spurious changes caused in functional connectivity due to head motion. This procedure was repeated for all the combinations of nuisance, motion, and spike regressors discussed previously, and the results compared for the high‐motion and the low‐motion subgroups.

### 2.7 Impact of head motion censoring threshold on the removal of motion‐induced artifacts

Motion censoring is a trade‐off between the quality and quantity of data. If PACE does correct for the lingering effects of spin history in the BOLD time series after the motion has ended, then a modest threshold with a small censoring window around the motion corrupted time points would provide us with a good compromise. Therefore, to understand the impact of motion censoring on the reduction of motion artifacts, we considered four cases of motion censoring using a lenient threshold of 0.5 mm and a stricter threshold of 0.2 mm: (a) Censoring of volumes whose FD_Power_ >0.5 mm and one volume after the motion corrupted volume (denoted as FD >0.5 mm, 0B + 1F, i.e., zero backward and one forward volumes are removed), (b) Censoring of volumes whose FD_Power_ >0.5 mm as well as one volume before and two volumes after the motion corrupted volume (FD >0.5 mm, 1B + 2F) (c) Censoring of volumes whose FD_Power_ >0.2 mm as well as one volume after the motion corrupted volume (FD >0.2 mm, 0B + 1F), and (d) Censoring of volumes whose FD_Power_ >0.2 mm as well as one volume before and two volumes after the motion corrupted volume (FD >0.5 mm, 1B + 2F). We examined both the motion‐BOLD relationships and the distance‐dependent connectivity artifact for each of the four cases to make a sound judgment on the appropriate motion threshold that prevents excessive loss of data, while still removing motion artifacts.

### 2.8 Calculation of RS‐fMRI based connectivity metrics

**Figure 1:**
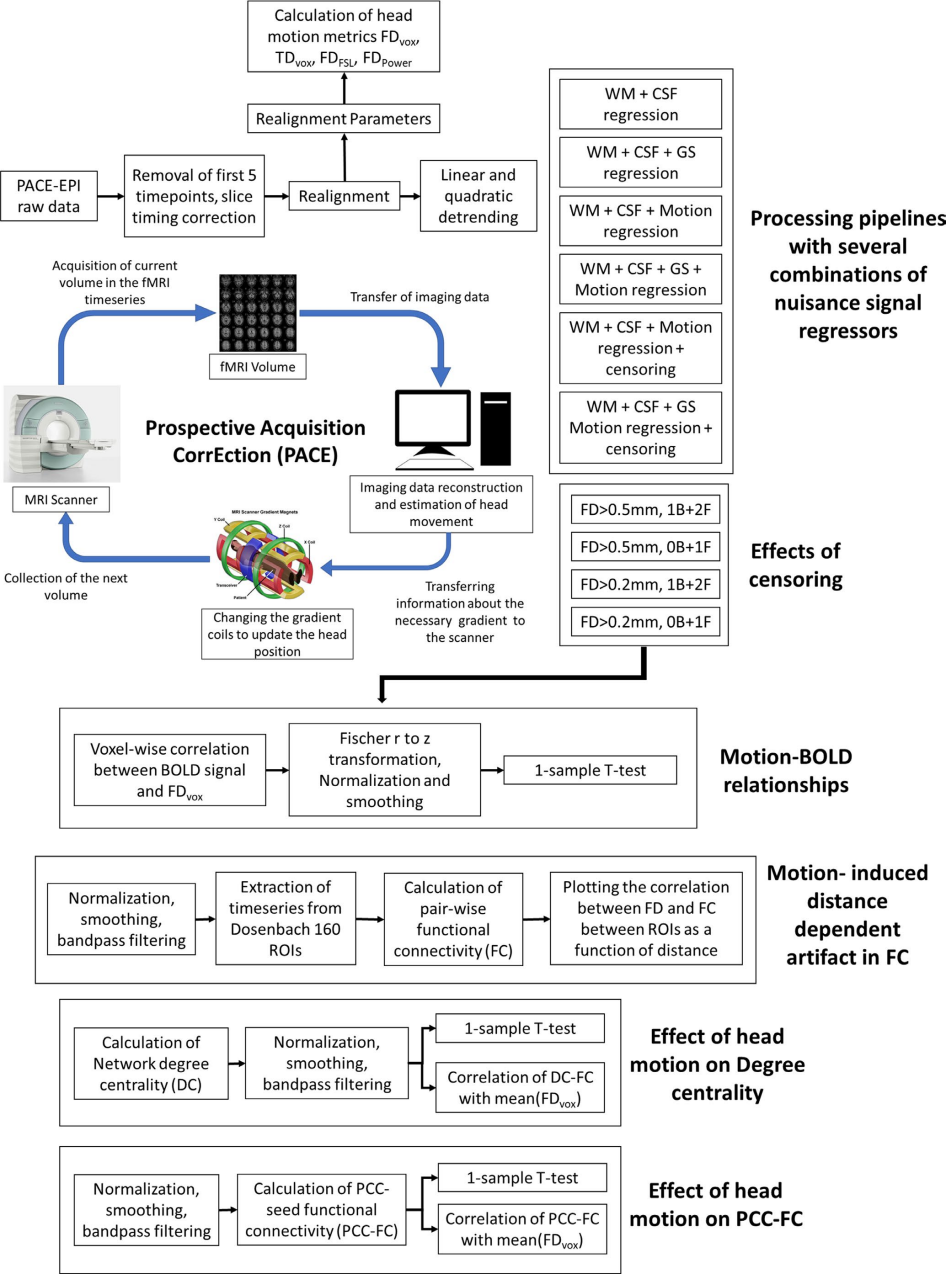
Flowchart summarizing our processing pipeline. Picture of the gradient coil taken from MRI: A Guided Tour, 2018 (Coyne, 2012). Reproduced with permission from the author. B, number of backward frames from the motion corrupted time points removed due to censoring; BOLD, blood‐oxygen‐level‐dependent signal; CSF, cerebrospinal fluid signal; EPI, echo‐planar imaging; F, number of forward frames from the motion corrupted time points removed due to censoring; FD, framewise displacement; GS, global signal; PCC, posterior cingulate cortex; TD, total displacement; WM, white matter signal

We calculated two RS‐fMRI based metrics: (a) Network DC and (b) PCC‐FC. These were chosen in order to (a) Evaluate the spatial relationship between functional connectivity metrics and motion and, (b) Compare the effectiveness of the motion correction strategies in high‐motion and low‐motion subgroups. Network DC was calculated as the weighted sum of significant positive connections for every voxel in the brain (Buckner, Andrews‐Hanna, & Schacter, 2008; Yan et al., 2013; Zuo et al., 2012). A connection was deemed significant if the correlation coefficient exceeded a threshold of 0.25 (*p* < 0.0001). A subject level *z*‐score was calculated by subtracting the mean for all voxels in the volume of a subject and dividing it by the standard deviation, to standardize the DC scores for group‐level analyses. These subject level z‐maps were registered to the MNI template and smoothed with a 4.5 mm^3^ Gaussian kernel. PCC‐FC was estimated by extracting the mean time series from the PCC: 0, −53, 26; diameter = 10 mm and then calculating the Pearson's correlation coefficient with other voxels in the brain as was done in other studies (Satterthwaite et al., 2013; Yan et al., 2013). This was done in the standardized space after preprocessing, filtering, normalization, and smoothing. These correlation values were transformed to *z* values using Fischer's *r* to *z* transformation. We calculated the correlation between the DC/PCC‐FC maps and FD_vox_ for each subject across every voxel to obtain maps indicating the relationships between motion and FC metrics for both high‐motion and low‐motion subgroups. To compare the effectiveness of the motion correction strategy across preprocessing pipelines with various nuisance regressors, we performed a *t* test for high‐motion and low‐motion subgroups separately. A flowchart summarizing our processing pipeline can be found in Figure 1.

## 3 RESULTS

### 3.1 Examination of BOLD time‐series data

**Figure 2:**
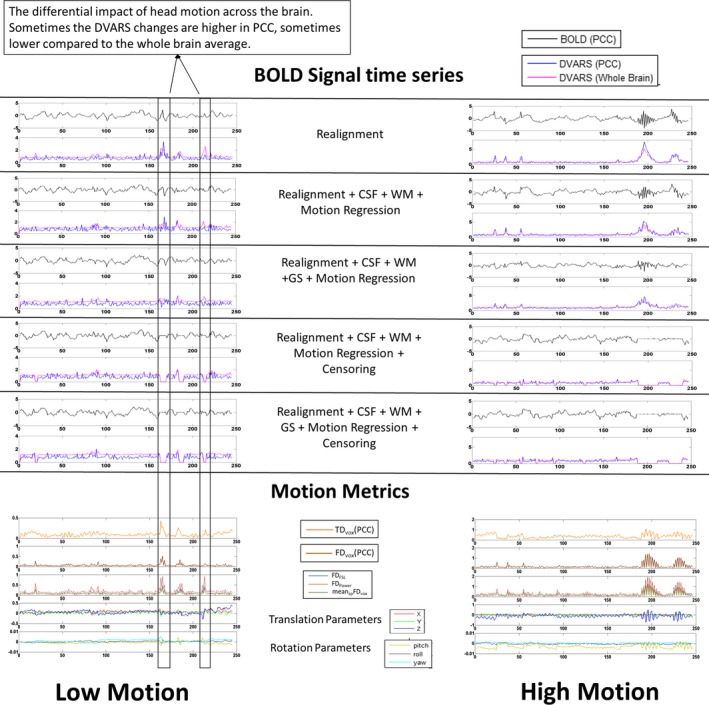
The PACE‐corrected time‐series extracted from the posterior cingulate cortex (PCC 0, −53, 26; 10 mm diameter sphere) at every step in the preprocessing pipeline for a representative subject in the high‐motion (right) and low‐motion (left) subgroups. Please note that the range of the y‐axis for both the groups is the same for blood‐oxygen‐level‐dependent (BOLD) time series and range from −5 to 5. However, for the motion metrics plots the range on the y‐axes are different in the left and right panels in order to better visualize the type of motion in low‐motion subjects. Large changes in the head position are associated with large changes in the BOLD signal. Regression of nuisance variables was not successful in eliminating large spikes in head motion in the high‐motion subject, but they were relatively successful in the low‐motion subject. The head motion can sometimes only selectively affect PCC, but not the whole brain. DVARS: Derivative of root mean squared variance over voxels. CSF, cerebrospinal fluid signal; FD, framewise displacement; FD_FSL_, FD calculated as described in Jenkinson et al. (2002); FD_Power_, FD calculated according to Power et al., (2012); FD_vox_, voxel‐specific framewise displacement calculated as detailed in Yan et al. (2013); GS, global signal; TD_vox_, voxel‐specific total displacement; WM, white matter signal

Figure 2 shows PACE‐corrected resting state BOLD time‐series extracted from the PCC in a representative high‐motion subject as well as a low‐motion subject and provides an insight into the effect of head motion on prospectively motion‐corrected BOLD signal. The effect of each of the preprocessing steps on the time courses of the BOLD signal (PCC), DVARS (PCC), and DVARS (whole brain), each of which are obtained from PACE‐corrected data, can be discerned. Figure 2 also displays motion metrics such as mean (TD_vox_ [PCC]) (TD of PCC), mean_sp_TD_vox_ (TD of the brain), mean (FD_vox_ [PCC]), mean_sp_FD_vox_, FD_FSL_, FD_Power_, and the six realignment parameters. There is a linear relationship between residual motion metrics, and FD_Power_ seems to have the highest of all the FD_vol_ measures and that FD_FSL_ and mean_sp_FD_vox_, closely align with each other.

**Figure 3:**
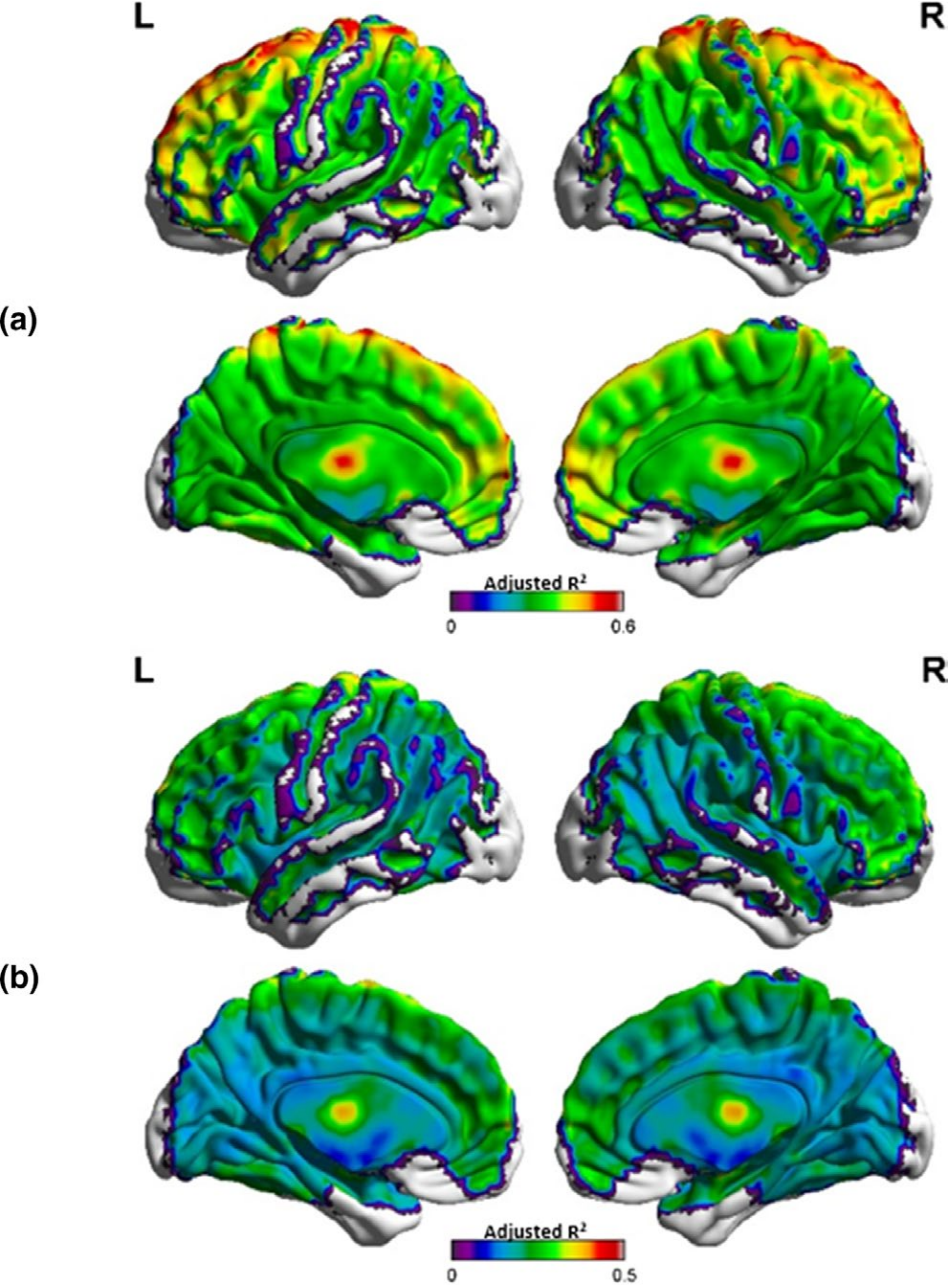
The average BOLD signal variance (adjusted *R* ^2^) explained by the 24 regressors used in the Friston‐24 motion regression model (a) and the six realignment parameters (b). Figures (a and b) are similar, except for that fact that 24 motion regressors (a) explain far more variance across the brain compared to using just six motion parameters (b). These motion regressors explain a modest amount of variance in the brain, with more variance explained in the frontal regions and less variance explained in other (especially posterior) regions. This is to be expected given that frontal regions experience more displacement than other regions of the brain Yan et al., (2013). BOLD, blood‐oxygen‐level‐dependent; L, left view; R, right view

Looking at the impact of the motion on the PACE‐corrected BOLD time series in Figure 2, we observe that large changes in the head position roughly correspond to the significant changes in the PACE‐corrected BOLD time series. While this has been shown to be true for non‐PACE data (Power et al., 2012, 2014; Satterthwaite et al., 2013), we show here that the same is true for PACE‐corrected data as well although the magnitude of such changes may be different in PACE data. DVARS, which measures the change in the BOLD signal, approximately follows the sharp rise and fall in head motion (as captured in the FD). It is also worth noting that some head movements were associated with larger signal changes in the PCC compared to the whole brain signal and in some cases, it was other way around. This points to the differential impact of head motion on the PACE‐corrected BOLD signal in different regions of the brain. In the high‐motion subject who was relatively still except for two large head movements, a ringing effect (rapid changes) in the PACE‐corrected BOLD signal and associated FD metrics can be observed. One possible reason for this could be that, due to the prospective correction by PACE, the scanner seems to be adjusting to the motion, thus causing a distinct effect on the BOLD time courses. Of course, these patterns appear to have reduced after WM, CSF, and GSR, and motion parameter regression but is still distinctly present after nuisance covariate regression, giving merit to the argument that censoring the motion corrupted volumes is the best way to eliminate the artifactual effects of residual head motion on the PACE‐corrected BOLD signal. Although the data are shown for just two subjects, it effectively demonstrates the limitations of nuisance signal regression in preprocessing rs‐fMRI data obtained with PACE. To understand how much variance is explained by the Friston‐24 motion parameters and the six realignment parameters, we estimated the average PACE‐corrected BOLD signal variance explained by the 24 motion parameters and six realignment parameters for each subject and averaged the results over all subjects as shown in Figure 3a,b, respectively. This result is pretty similar to figure 1B in Satterthwaite et al., 2013, who used adjusted *R* ^2^ maps to illustrate the signal varaince explained by using six realignment parameters. The BOLD signal from regions which are farthest from the center of the brain are affected the most by head motion and consequntly more variance in the PACE‐corrected BOLD signal is explained by the 24 motion parameters. Also, compared to six realignment parameters (Figure 3b) use of 24 motion parameters (Figure 3a) explained a lot more variance (as observed by their adjusted *R* ^2^ values) across the brain.

### 3.2 Voxel‐wise relationships between FD and PACE‐corrected BOLD signal

**Figure 4:**
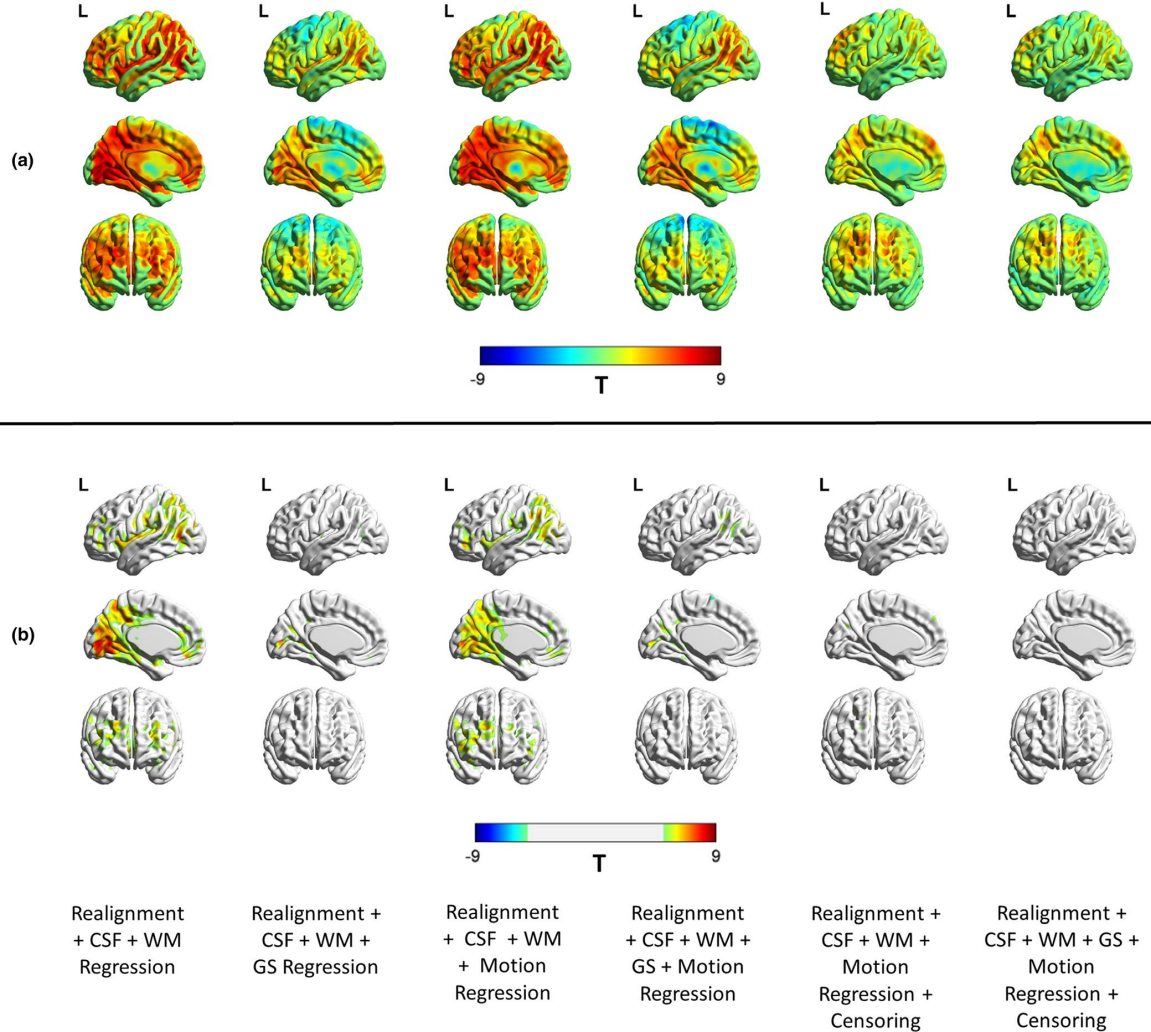
Illustration of the reduction in the relationship between motion and PACE‐corrected BOLD data for different nuisance variable regressors. The unthresholded T‐maps are shown in (a) and the thresholded (*p* < 0.05, FDR corrected) maps are shown in (b). The results indicate that motion regression did not remove motion‐BOLD relationships visibly. However, GS regression did seem to reduce these relationships, with some regions now showing a negative correlation. (b) After the nuisance variance regressions, some regions did exhibit significant positive relationships with the BOLD signal, though no negative relationships remained. With censoring, both positive and negative relationships are almost absent. BOLD, blood‐oxygen‐level‐dependent; CSF, cerebrospinal fluid signal; GS, global signal; L, left view; PACE, Prospective Acquisition CorrEction; WM, white matter signal

The sensitivity of the BOLD time series to head motion artifacts is spatially varying and this can be characterized by the FD‐BOLD relationship. Consistent linear relationships (or correlations) between FD_vox_ and the BOLD signal across the brain is indicative of the motion artifact and can affect the estimation of functional connectivity between brain regions. Figure 4 shows the raw FD_vox_‐BOLD correlation maps as well as maps thresholded at (T > 4.95, *p* < 0.05, FDR corrected). As was observed with the PACE‐corrected time‐series data, WM, CSF, and motion regression did not remove significant motion‐BOLD relationships. An interesting observation is that negative motion‐BOLD relationships which are associated with large head movements (Yan et al., 2013) were absent in the thresholded maps obtained from PACE‐corrected data. It can be seen from Figure 4 that none of the voxels exceed the negative threshold, implying no significant negative motion‐BOLD relationships were present. It is noteworthy that results from non PACE‐corrected data reported before show significant negative motion‐BOLD relationships (Yan et al., 2013). With the addition of GSR, the large positive relationships were reduced across the brain, but negative correlations were introduced. However, it should be noted that none of the negative motion‐BOLD correlations were significant. With relatively modest motion censoring (FD >0.5 mm, 1B + 2F) and without GSR, motion BOLD relationships were not significant (*p* > 0.05), and very few positive relationships survived the thresholds. In order to obtain an equivalent result with non‐PACE data, Yan et al., had to use a much stricter censoring threshold of FD_Power_ >0.2 mm coupled with GSR (Yan et al., 2013). If we used GSR or increased the censoring threshold to those used by Yan et al., 2013, all motion‐BOLD relationships were eliminated. This indicates that one could use liberal censoring (thereby retaining more data) and avoid the confounding effects of GSR and yet eliminate all negative, and most positive motion‐BOLD relationships using PACE data.

**Figure 5:**
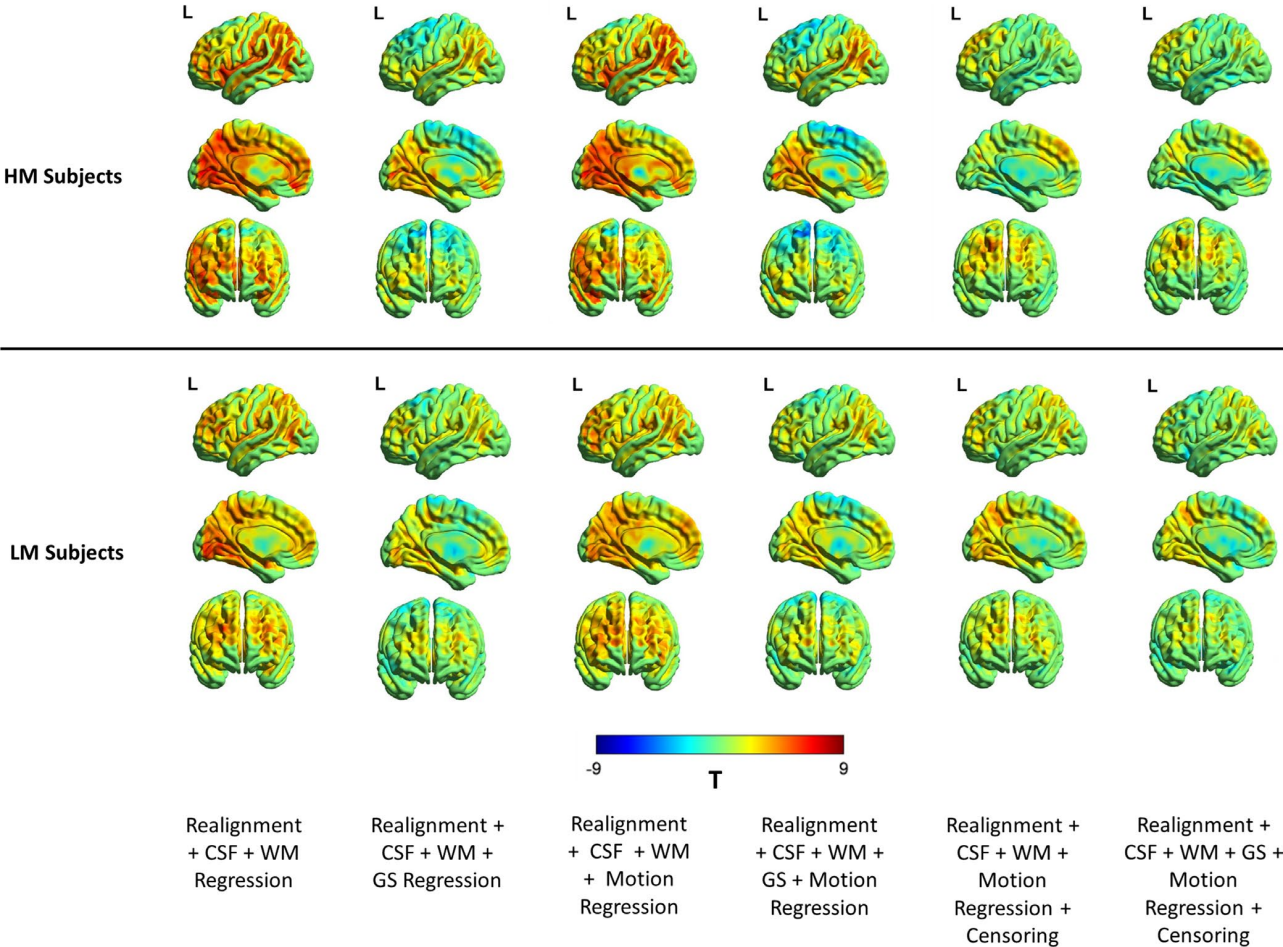
The unthresholded T‐maps illustrating the relationship between the PACE‐corrected BOLD signal and voxel‐specific framewise displacement for the high‐motion and the low‐motion subgroups. Cerebrospinal fluid, white matter, and motion regression are relatively ineffective in reducing the motion‐BOLD relationships both in high‐motion and low‐motion subjects. Large motion‐BOLD relationships are comparatively fewer in low‐motion subjects, as expected. Global signal regression significantly increased negative motion‐BOLD relationships in high‐motion subgroup, but not by much in the low‐motion subgroup. With motion censoring, GSR has a relatively negligible effect on the motion‐BOLD relationships in both the subgroups. BOLD, blood‐oxygen‐level‐dependent; CSF, cerebrospinal fluid signal; GS, global signal; HM, high‐motion cohort; L, left view; LM, low‐motion cohort; PACE, Prospective Acquisition CorrEction; WM, white matter signal

**Figure 6:**
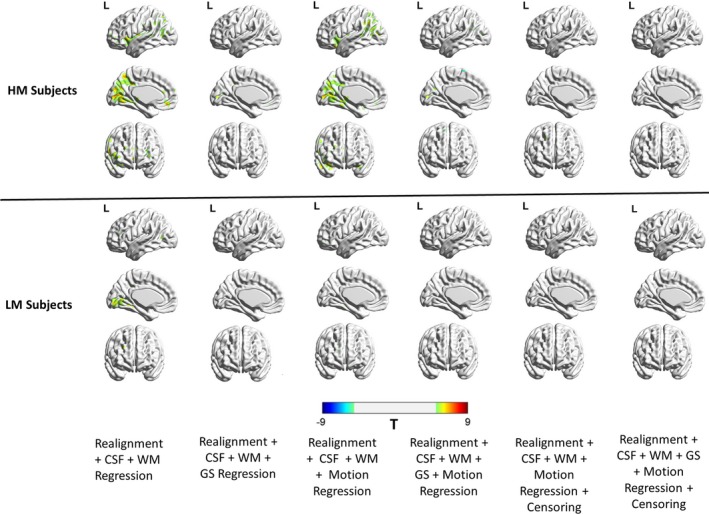
Thresholded correlation maps between the PACE‐corrected BOLD signal and voxel‐specific framewise displacement across the brain. The figure shows the relative absence of significant (*p* < 0.05, FDR corrected) motion‐BOLD relationships in low‐motion subjects compared to the high‐motion subjects. The reduction in motion‐BOLD relationships after GS and motion regression in high‐motion subjects is stark, although residual correlations in the visual cortex are only eliminated after motion censoring. BOLD, blood‐oxygen‐level‐dependent; CSF, cerebrospinal fluid signal; GS, global signal; HM, high‐motion cohort; L, left view; LM, low‐motion cohort; PACE, Prospective Acquisition CorrEction; WM, white matter signal

To better understand the patterns of these relationships between high‐motion and low‐motion subjects, we repeated the analysis separately for high‐motion and low‐motion subgroups. The result is shown in Figure 5 and the corresponding thresholded T‐maps for high‐ and low‐motion subgroups shown in Figure 6. The results show small motion‐BOLD correlations for low‐motion subgroup as expected, with significant correlations (*p* < 0.05, FDR corrected) only restricted to the visual areas after WM and CSF regression. Further steps of preprocessing eliminated even those correlations to below significance. However, the relationships for high‐motion subgroup relationships reduced to below chance levels in most areas only after motion censoring. Qualitatively, motion‐BOLD correlations obtained from PACE data appear to be smaller in magnitude and spatial extent when compared to those obtained from non‐PACE data (in both low‐ and high‐motion subjects) reported previously (Yan et al., 2013).

### 3.3 Motion‐induced distance‐dependent artifact in resting‐state functional connectivity

**Figure 7:**
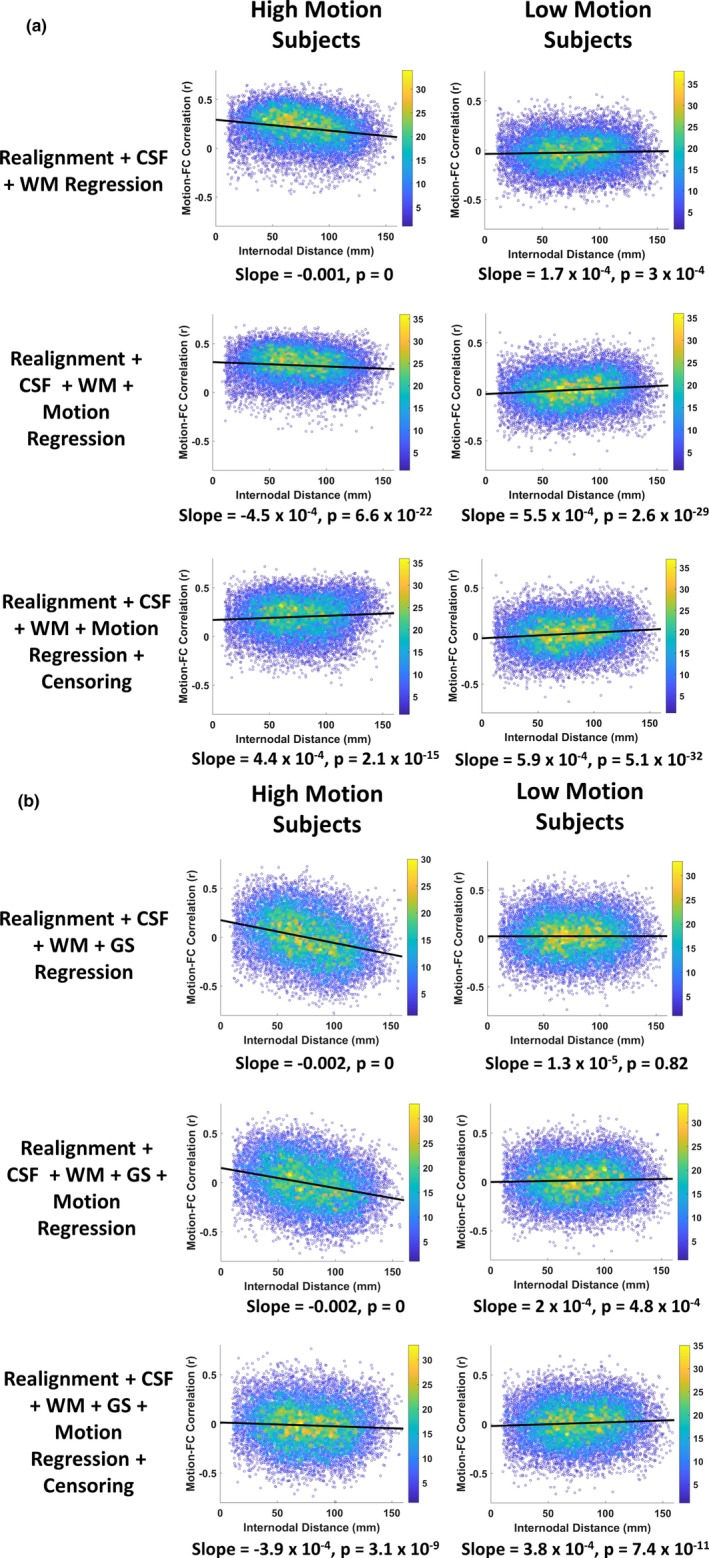
The figure shows the framewise displacement–resting state functional connectivity (FD‐RSFC) correlations for subjects in the high‐ and low‐motion subgroups (a) without global signal regression (GSR), and (b) with GSR. The motion‐induced distance‐dependent RSFC artifact is almost absent in the low‐motion subgroup for all stages and all combinations of nuisance signal regression. The color bar on the right indicates the density of points. The high‐motion subgroup does show the artifact which is only reduced after motion censoring. GSR distorts the FD‐RSFC relationships significantly, especially in the high‐motion subgroup, though after motion censoring, the data are relatively free from the artifact in both the subgroups, with and without GSR. CSF, cerebrospinal fluid signal; GS, global signal; WM, white matter signal

When functional connectivity is estimated with motion‐corrupted data, connectivity strengths between two brain regions can be dependent on the relative location of the regions and the similarity in magnitude and the direction of the displacement experienced by head motion. This artifact helps us in evaluating the success of a motion correction strategy and the absence of the motion artifact in the data. We plot the 12,720 connectivity values (obtained from PACE‐corrected BOLD time series) which were correlated with each subject's summary head motion (mean [FD_FSL_]) as a function of distance. This was done for all combination of nuisance regressors and motion censoring, and we show the results for the high‐motion and the low‐motion subgroups separately in Figure 7. Ideally, if head motion was not artifactually modulating the connectivity values, we expect the plot to be a flat (zero slope) line and a zero intercept, since there must be no relationship between most connectivity paths and head motion. But as Figure 7 illustrates, the distance‐dependent artifact was present for all combinations of nuisance variable regression including WM, CSF, GS, and Friston‐24 motion regression. The correlation of head movement with the connectivity metrics exhibited positive values for all distances and only with the introduction of GSR, were the correlation with motion became negative for functional connectivity between farther regions. With motion censoring, this artifact did not seem to have been completely eliminated, especially in high‐motion subjects, with a small slope and a positive intercept when fitted by a linear trend line. There was a positive correlation between FC and head motion at all distances in high‐motion subjects. The artifact almost seems absent in low‐motion subjects for all combinations of nuisance variable regression and censoring as the slope is small and the line is relatively flat. In contrast, previous reports with non‐PACE data indicate that the distance‐dependent artifact could not be eliminated (unless censoring thresholds were more severe than what we have used) even in low‐motion subjects (Satterthwaite et al., 2012). A few more observations from the figure are that GSR appears to distort the distance‐dependent artifact and makes the artifact worse by increasing the slope in high‐motion subjects and the variance in low‐motion subjects. This result is in agreement with the observations made by Jo et al. that GSR distorts functional connectivity values (Jo et al., 2013). However, when GSR was combined with censoring, it did seem to eliminate the distance‐dependent artifact even in subjects with high motion. Since we used a relatively modest threshold of 0.5 mm with a censoring window of one previous volume and two forward volumes, we wanted to see if a more severe threshold of FD_Power_ >0.2 mm, would have any additional benefits at the cost of substantial loss of data.

### 3.4 Impact of censoring threshold on the existence of motion artifacts

**Figure 8:**
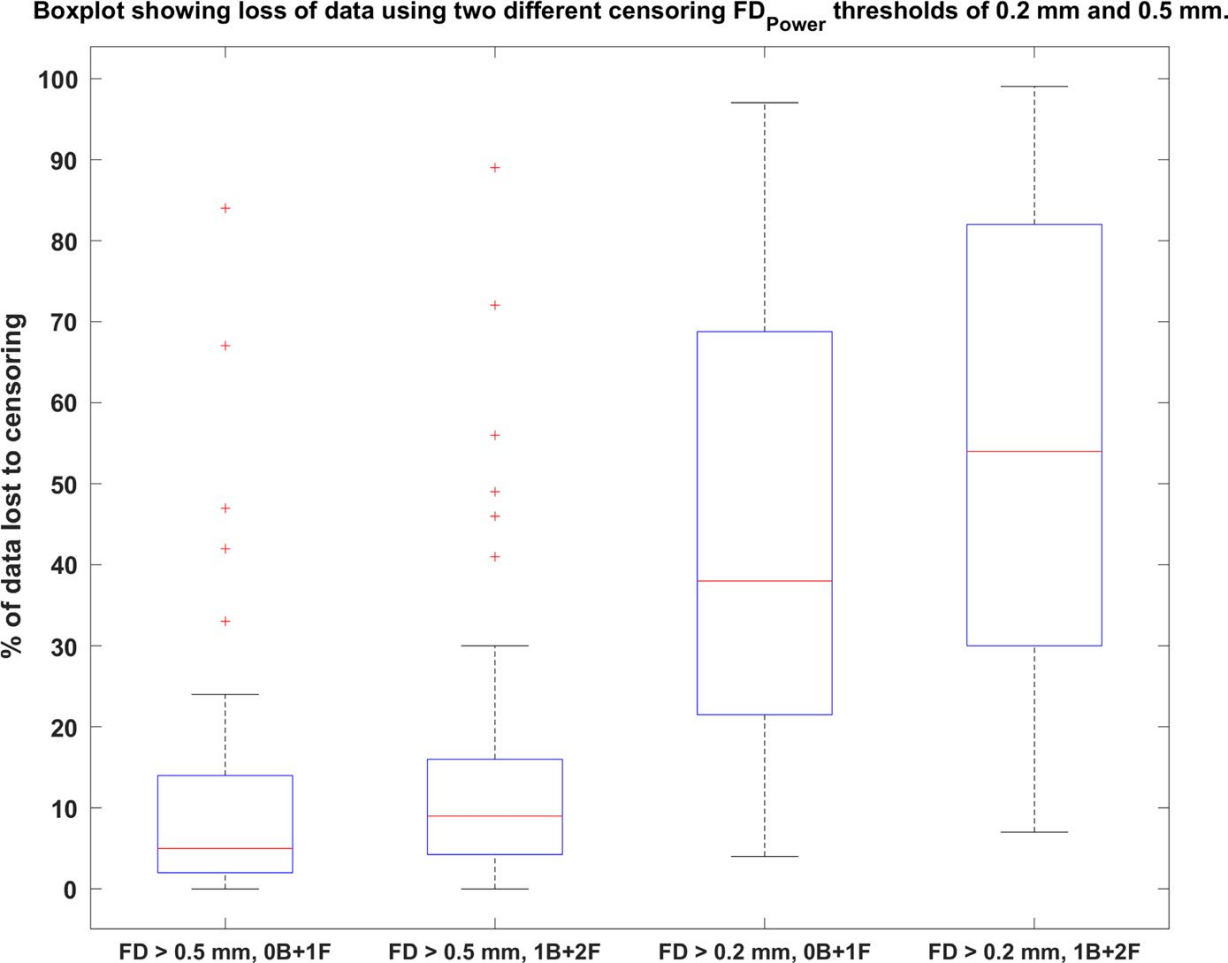
A boxplot shows the percent loss of data for four different censoring scenarios with framewise displacement (FD_Power_) used to quantify head motion. In the four scenarios a head motion threshold (FD_Power_ either >0.2 mm or >0.5 mm) was used to mark time points corrupted with head motion, and the time point, along with either one forward (0B + 1F) or two forward and one backward (1B + 2F), were also removed with the motion corrupted time points. The boxplot indicates a large loss of data, with data from many subjects completely unusable when using a stricter censoring threshold (FD_Power_ >0.2 mm), compared to a more lenient threshold (FD_Power_ >0.5 mm).

**Figure 9:**
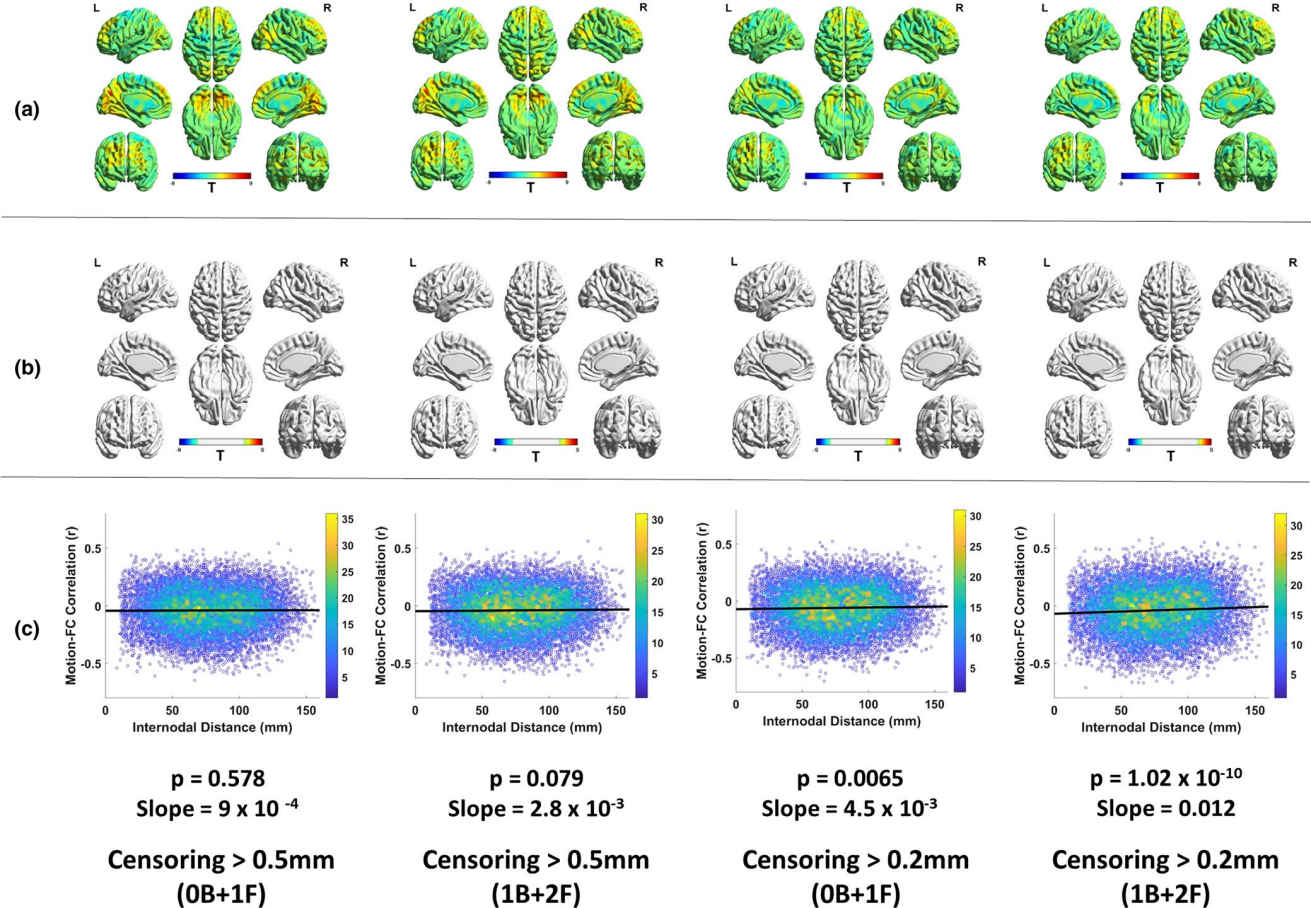
The absence of motion artifacts for the four cases of motion censoring. (a) The motion‐BOLD relationships indicate very small positive motion‐BOLD relationships in the visual cortex, which are removed by censoring the volumes at a lower (more stringent) threshold. (b) Thresholded motion‐BOLD relationships for the figures shown in (a). It must be noted that none of the volumes exhibited significant correlations for all the four scenarios of censoring. (c) The framewise displacement—resting state functional connectivity correlations, which can be used to detect the presence of the motion‐induced distance‐dependent functional connectivity artifact, shows that for all the cases of censoring, the artifact was absent. The color bar on the right indicates the density of points. A stricter threshold for censoring or a larger censoring window does not seem to have a detectable improvement in data quality. When taken in light of findings from the Figure 8, it appears that PACE, when combined with retrospective motion correction allows us to obtain same quality data with a more liberal threshold, thereby saving data. B, number of backward frames from the motion corrupted time points removed due to censoring; BOLD, blood‐oxygen‐level‐dependent; F, number of forward frames from the motion corrupted time points removed due to censoring; L, left view; R, right view

In order to better understand the dynamics between the removal of motion artifacts and preservation of noncorrupted PACE‐corrected BOLD data, we experimented by using two different censoring thresholds (0.5 and 0.2 mm) and two censoring windows around the motion corrupted volumes (0B + 1F and 1B + 2F). This gave rise to four scenarios of motion censoring (a) FD_Power_ >0.5, 0B + 1F, (b) FD_Power_ >0.5, 1B + 2F, (c) FD_Power_ >0.2, 0B + 1F, and (d) FD_Power_ >0.2, 1B + 2F. Figure 8 shows the fraction of time points marked for excessive head motion and removed for each of the above censoring scenarios. As expected, there was huge loss in data when we used censoring at 0.2 mm compared to 0.5 mm. In fact, the number of subjects, who had at least 3 min of good data or 180 time points in our case was reduced from 47 to 24, when the threshold was greater than 0.2 mm and one volume before and two volumes after the motion were removed. We used the presence of the significant (*p* < 0.05, FDR corrected) motion‐BOLD relationships and the existence of the motion‐induced distance‐dependent functional connectivity artifact (FD‐RSFC correlations) to assess the quality of nonmotion corrupted data. To be fair in the comparison, we used the same 24 remaining subjects for all the four censoring cases. Since most of the subjects left had pretty low motion, we expected results similar to those obtained by the low‐motion dataset. It is noteworthy that several subjects were common to both the subsets of data and there was relative absence of motion artifacts in the low‐motion subgroup even with relatively less preprocessing. The unthresholded T‐maps are shown in Figure 9a and the thresholded T‐maps (*p* < 0.05, FDR corrected) are shown in Figure 9b. As Figure 9b shows, motion‐BOLD relationships were below significance for all voxels in all the four scenarios of motion censoring, though the values of few motion‐BOLD relationships in the visual areas are removed with the more stringent threshold. The motion‐induced distance‐dependent connectivity artifact appeared to be appeared to be considerably reduced (Figure 9c) in the four cases as the slope was very small. But the slope was not significant (*p* > 0.05) for the case with censoring FD_Power_ >0.5, 0B + 1F. The slope was small as well as significant (*p* < 0.05) for other censoring scenarios, indicating that motion‐induced distance‐dependent connectivity artifact is eliminated after censoring the data at FD_Power_ >0.5, 1B + 2F. Increasing the censoring window size beyond the motion corrupted volume and a single volume after the corrupted volume, did not seem to have any effect on the data even after filtering the time series. Our results indicate that censoring volumes at a more stringent threshold of 0.2 mm or increasing the censoring window size to include more volumes did not have a detectable improvement in the data quality as the artifacts were almost eliminated at 0.5 mm, but it came at the cost of substantial loss of data. This indicates that PACE when combined with retrospective motion correction methods including motion censoring at 0.5 mm was effective in removing head motion artifacts while still saving data.

### 3.5 Impact of motion on functional connectivity estimates of DC and PCC‐FC

**Figure 10:**
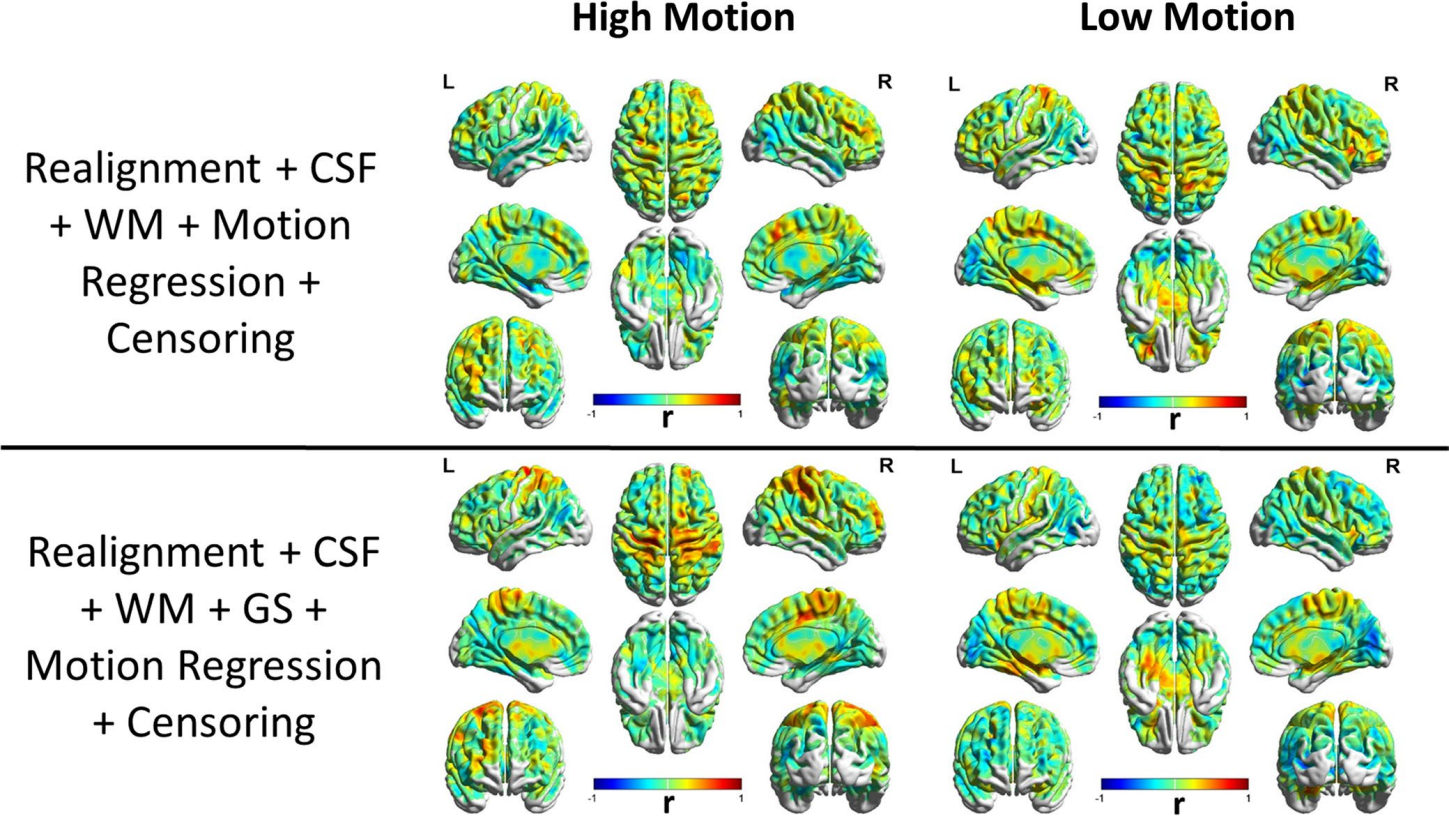
Unthresholded spatial map of the Pearson's correlation coefficient between the degree centrality obtained from PACE‐corrected BOLD data and residual head motion as captured by mean voxel‐wise framewise displacement across subjects, shown for subjects in high‐motion (left) and low‐motion (right) groups separately. Large positive correlations were observed in the sensorimotor cortex in the low‐motion subgroup as well as in the high‐motion subgroup with nuisance variable regression and censoring. This illustrates that some changes in functional connectivity might have a neural origin and it could be confounded with changes due to motion artifact as even motion artifact causes changes in functional connectivity. BOLD, blood‐oxygen‐level‐dependent; CSF, cerebrospinal fluid signal; GS, global signal; L, left view; PACE, Prospective Acquisition CorrEction; R, right view; WM, white matter signal;

**Figure 11:**
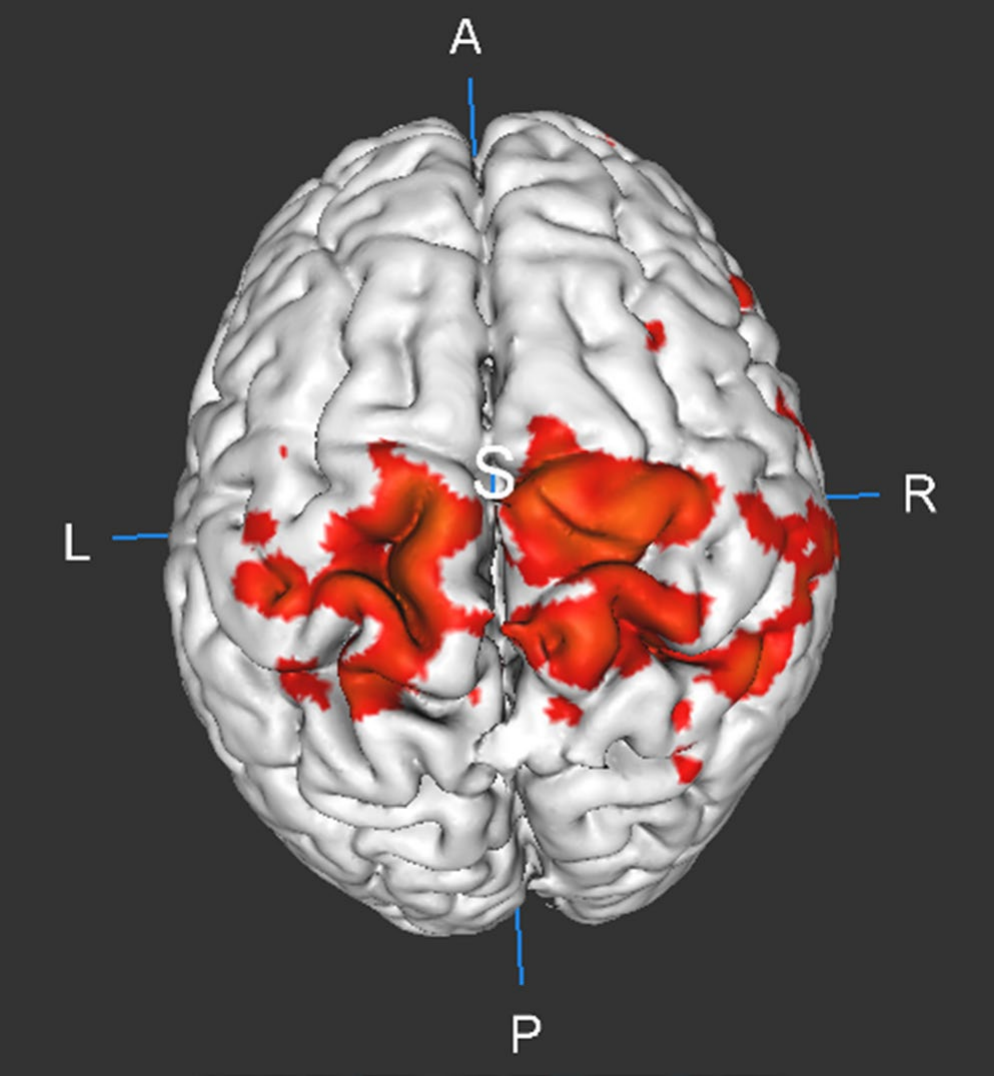
Figure showing the thresholded (*p* < 0.05) correlation (R) map of network degree centrality (DC) with head motion of the brain after nuisance variable regression including CSF, white matter signal, Friston‐24 motion regression and motion censoring in all the subjects. Significant positive correlations can be observed between residual head motion in PACE‐corrected data and DC in the sensorimotor cortex. This shows that DC in the sensorimotor could possibly be attributed to neural processes responsible for head motion. A, anterior; CSF, cerebrospinal fluid signal; L, left; P, posterior; PACE, Prospective Acquisition CorrEction; R, right; S, superior

**Figure 12:**
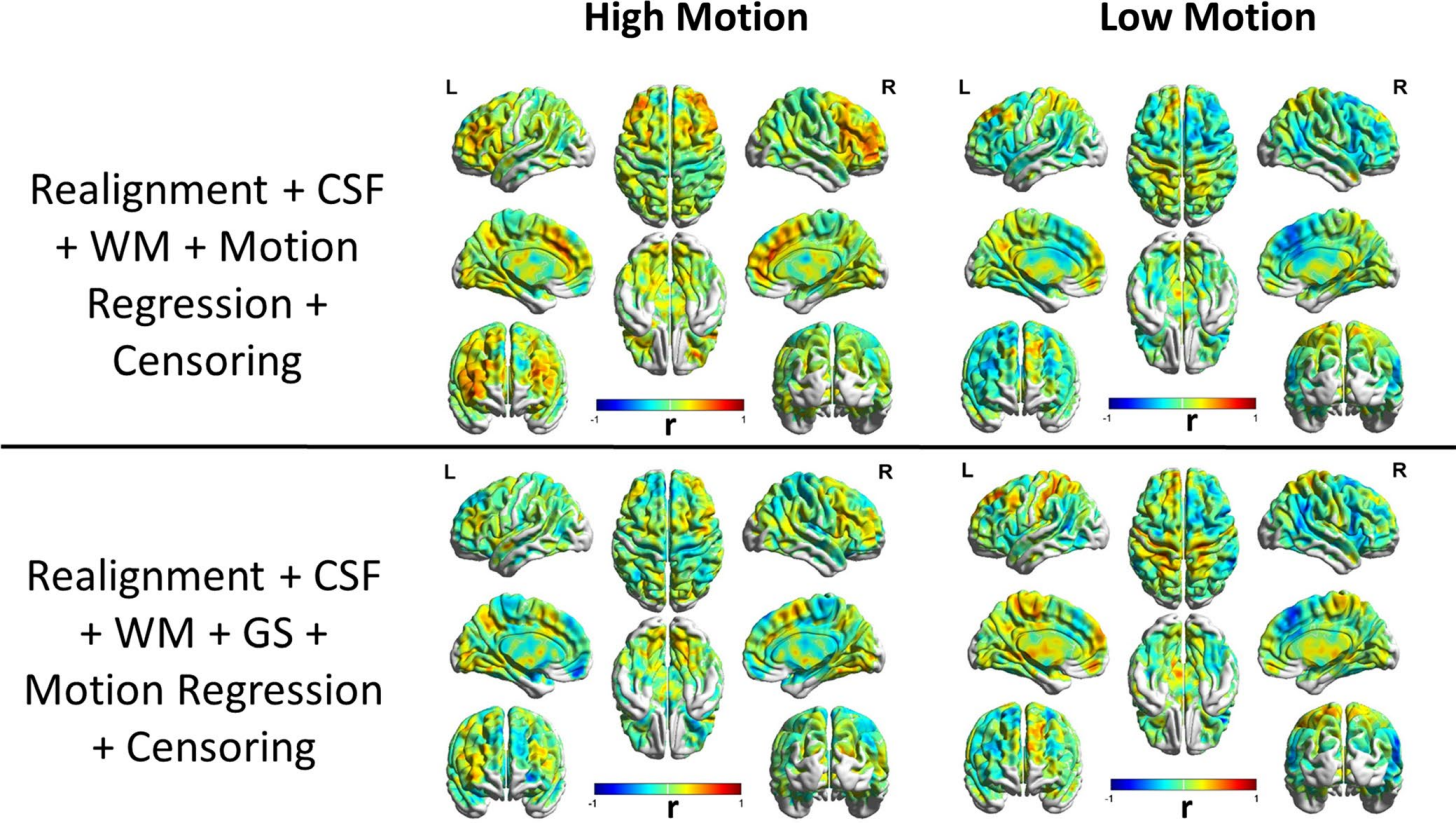
Correlation between seed‐based functional connectivity of posterior cingulate cortex and head motion (as captured by mean voxel‐wise framewise displacement across subjects) shown for subjects with high head motion (left) and low head motion (right) groups separately. Large correlations were observed across the brain in both low‐ and high‐motion subgroups. With motion censoring and global signal regression, the correlations in the high‐motion group were reduced. This illustrates their relative effectiveness in reducing motion artifacts particularly in subjects with high head motion. CSF, cerebrospinal fluid signal; GS, global signal; L, left view; R, right view; WM, white matter signal

As seen earlier, motion does affect functional connectivity and other measures derived from it even with EPI‐PACE acquisition. In order to understand the residual relationships between functional connectivity metrics and motion, we calculated the Pearson's correlation coefficient between head motion, that is, mean [FD_vox_], and DC (Figure 10) separately for the high‐motion and the low‐motion subgroups. As shown in Figure 10, DC was relatively robust to the influence of motion artifact due to *Z*‐standardization (Yan et al., 2013). This implies that nuisance variable regression and censoring did not have much impact on the FD‐DC correlations. However, we found large positive correlations in the sensorimotor cortex, and the correlations increased after motion artifacts were removed from the data via motion regression and censoring. A more detailed image of motion‐DC correlation after in the sensorimotor cortex, after thresholding at (*p* < 0.05, FDR corrected) is shown in Figure 11. This effect was observed both in the high‐motion and the low‐motion subgroups. A similar result was reported by Pujol et al., indicating that there is a component of motion‐related connectivity changes that may have a neural basis and may not be just a consequence of the motion artifact (Pujol et al., 2014). The FD‐PCC functional connectivity correlation map (shown in Figure 12) identifies the regions whose correlation with PCC varies as a function of subject head motion. We observed significant (*p* < 0.05, FDR corrected) negative correlations between residual motion and PCC‐FC in the frontal regions in the low‐motion subgroup and a significant reduction in the positive correlations especially in subjects with high motion as GS, motion regression, and censoring were performed. This highlights their relative effectiveness in reducing motion artifacts, particularly in subjects with high head motion.

**Figure 13:**
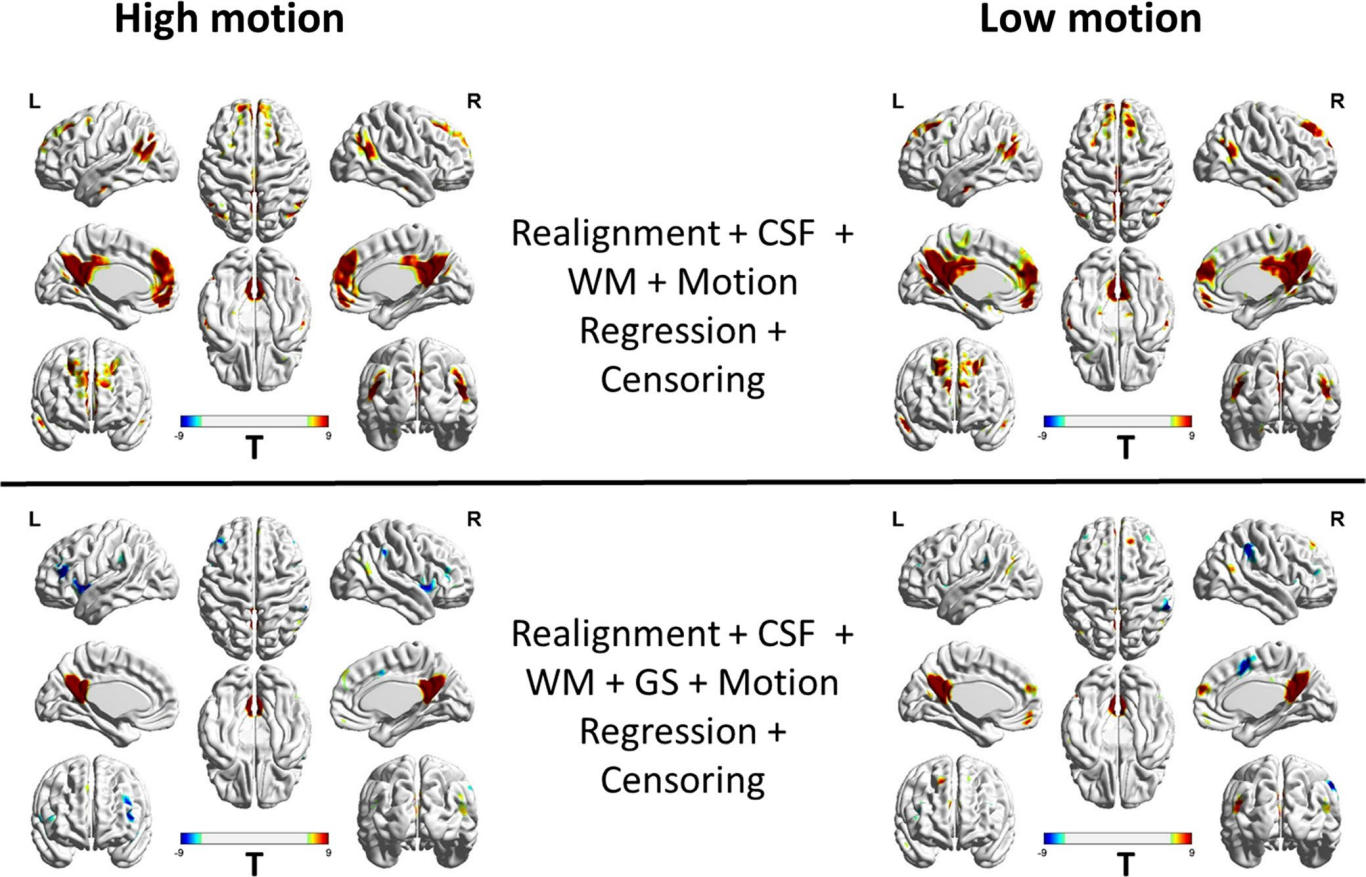
A comparison of regions with significant correlations (*p* < 0.05, FDR corrected) with posterior cingulate cortex (PCC: 0, −53, 26; 10 mm diameter sphere) as the seed region PCC‐functional connectivity. This figure is shown for both high‐motion (left) and low‐motion subjects (rights). With the addition of global signal regression (GSR), anticorrelated networks were observed. In high‐motion subjects with GSR, the correlation between medial prefrontal cortex and PCC was reduced to chance levels, while it was still present in the low‐motion subjects. This illustrates that GSR is also likely removing neural components along with motion‐induced noise signal. CSF, cerebrospinal fluid signal; GS, global signal; L, left view; R, right view; WM, white matter signal

A two‐tailed *t* test was performed across subjects in the high‐ and low‐motion subgroups separately by using individual subject DC maps as the sample to find consistent patterns across the motion subgroups (results not shown). This further demonstrates that DC is robust to various motion correction strategies, and similar results can be obtained with different motion populations. In Figure 13, we show a similar result with PCC seed‐based functional connectivity. The regions commonly associated with the default mode network (DMN) were observed in the PCC seed‐based FC map including regions such as the medial prefrontal cortex (mPFC), inferior parietal lobe, and lateral temporal cortex, without the GSR (Buckner et al., 2008; Koshino, Minamoto, Yaoi, Osaka, & Osaka, 2014; Raichle et al., 2001). But with GSR, anticorrelated and task‐positive networks such as the dorsal attention system and the hippocampal‐cortical memory system were observed as expected (Fox, Zhang, Snyder, & Raichle, 2009). However, it is important to note that in the high‐motion subjects with GSR, the mPFC which is an integral part of DMN, was absent, whereas it was present in the low‐motion subjects even after GSR. This illustrates that GSR is also likely removing neural components along with motion‐induced artifacts. Other than mPFC, other significant regions were commonly found in both the low‐motion and the high‐motion subgroups. Therefore, care must be taken when GSR is used in the preprocessing pipeline in the context of PACE‐corrected BOLD data as well.

## 4 DISCUSSION

This section is organized as follows. First, we discuss the principal advantages of using PACE in combination with retrospective motion correction strategies for controlling head motion artifacts in resting state fMRI data. Next, we discuss the effectiveness of various retrospective motion correction strategies when used in combination with PACE. Subsequently, we discuss identifying and separating neural correlates of head motion from motion artifacts using deconvolved PACE‐corrected BOLD data. This is followed by a discussion of other potential retrospective motion correction strategies which might be beneficial when used in combination with PACE, but which we have not been investigated here. Finally, we discuss some limitations of the current study which need to be kept in mind while interpreting our results.

### 4.1 The principal advantages of prospective motion correction (PACE)

In this study, we examined the effectiveness of PACE in reducing motion artifacts in resting state fMRI data. In combination with the retrospective motion correction methods, using PACE‐corrected EPI sequence eliminated most of the motion artifacts. Specifically, we found that PACE provides two primary advantages over conventional EPI sequences. First, PACE was effective in eliminating significant negative motion‐BOLD relationships. Significant voxel‐wise negative motion‐BOLD relationships are typically associated with large signal dropouts caused by relatively large head motion (Satterthwaite et al., 2013; Yan et al., 2013) when scanned with a typical EPI sequence. Given the general difficulty in reducing these negative motion‐BOLD relationships, PACE may provide a solution to this issue. However, these results should be interpreted with caution as the relatively small sample sizes of our data compared to the large sample sizes of previous studies could limit the utility of such direct comparisons. Second, previous reports have suggested a stringent censoring threshold (FD_Power_ >0.2 mm) for satisfactorily controlling the level of motion artifacts in resting state fMRI data (Power et al., 2014; Satterthwaite et al., 2013; Yan et al., 2013). However, with PACE, we found that censoring with a less stringent threshold (FD_Power_ >0.5 mm) and a smaller window around the motion corrupted time point, provided qualitatively equivalent reductions in the motion artifact. Unfortunately, it is very difficult to directly compare our results on PACE‐EPI with those on typical EPI sequences, as the same subjects were not scanned with typical EPI sequences. With that caveat in mind, using a liberal censoring strategy, we were able to reduce motion artifacts to almost chance levels even in subjects with relatively large residual head motion. This will likely provide significant savings in data which would otherwise be lost to censoring. Given this scenario, acquiring data with EPI‐PACE might result in larger amount of usable data and hence more robust analyses. The EPI‐PACE data used for analysis and processing in this paper is being made publicly available (Lanka & Deshpande, 2018). We invite other researchers to contribute EPI‐PACE resting state fMRI data to this sample so that our inferences can be verified in a larger cohort.

### 4.2 Effectiveness of retrospective motion correction methods when used in combination with PACE

Since PACE is a prospective motion correction sequence, the best advances in retrospective motion correction can still be used with equal or greater effectiveness when they are combined with PACE. The motion parameters captured are residual motion parameters after motion correction by PACE, not the actual subject motion. The ability of CSF and WM regression in removing the motion‐induced signal variance in resting state fMRI data is limited as reported by previous studies (Satterthwaite et al., 2013; Yan et al., 2013), a fact confirmed by our results. We used motion regression by the Friston‐24 model, which was shown to be the best performing model previously (Satterthwaite et al., 2013; Yan et al., 2013) and our results confirm the same. Obviously, higher‐order motion models might explain larger amount of variance for high‐motion datasets across the brain, but it comes at the cost of significant loss of degrees of freedom and result in a drop in the BOLD sensitivity (Beall & Lowe, 2014).

Although several previous studies recommend the use of GSR for reducing motion artifacts (Power et al., 2012, 2014; Pujol et al., 2014; Yan et al., 2013), the effectiveness of GSR in reducing motion artifacts in the BOLD signal as well as lowering FD‐RSFC correlations is mixed. Our results (Figure 4) are in agreement with the previous studies indicating that GSR effectively reduces the positive motion‐BOLD relationships but increases the negative motion‐BOLD relationships (Yan et al., 2013). GSR also distorted the FD‐RSFC correlations (Figure 7) considerably (Jo et al., 2013). GSR reduced the functional connectivity of mPFC with the PCC seed (Figure 13), a key component of the DMN, in the high‐motion subgroup. Other issues with GSR include the fact that it distorts the distribution of correlation values (Murphy et al., 2009), and could alter interindividual differences at the group level (Gotts et al., 2013; Saad et al., 2012). Given that PACE provides an additional strategy for motion correction, it could be used without GSR to achieve better quality data compared to conventional EPI coupled with no GSR. On the other hand, for the proponents of GSR, PACE's tendency to remove negative motion‐BOLD relationships may at least partially cancel out the negative motion‐BOLD relationships introduced by GSR.

We found censoring high‐motion time points from the data to be the most effective retrospective motion correction. With censoring, spurious motion‐BOLD relationships (Figure 6) and distance‐dependent functional connectivity artifacts (Figure 7) were almost eliminated in high‐motion subjects. An extremely important issue, when performing censoring is to determine how much resting state data is sufficient for stable and reliable estimation of resting‐state functional connectivity (RSFC) metrics. Some have suggested at least 4 min (Satterthwaite et al., 2013) and others believe that 3 min of RS‐fMRI data to be sufficient (Yan et al., 2013). While comparing usable data available after censoring (Figure 8), we have assumed that one has to have at least 3 min of data. In addition to scan time, the sampling period (TR) is also an important consideration. The value of the FD, used for identifying motion corrupted time points is paramount while censoring as it is heavily dependent on TR. Sampling the brain at a smaller TR tends to divide larger motion into smaller components, hence might have different effects on the presence of motion artifacts and motion correction. Also, censoring alters the temporal structure of the data even if the censored time points are interpolated. This affects frequency‐based analyses, moving window‐based dynamic functional connectivity (DFC), and effective connectivity (EC) calculations. So, all analyses which require an intact temporal structure of the data might want to avoid censoring. In such cases, PACE offers a way of obtaining relatively cleaner data without censoring, although motion artifacts cannot be completely eliminated without at least liberal censoring even when using PACE.

The effectiveness of group‐level motion correction by including individual motion estimates in group‐level analyses has been reported before (Power et al., 2014; Yan et al., 2013). Group‐level regression with individual motion estimates effectively removes potential motion‐related artifacts but may also remove changes related to neural activity (Pujol et al., 2014). Many pathological conditions are associated with changes in regional functional connectivity. These changes in connectivity might be biased by the group effects of the subject head motion especially in hyperkinetic populations. So, it might be difficult to separate motion artifacts from disease effects, especially since the effect of interest is correlated with head motion. Unfortunately, in these cases group‐level motion correction cannot be performed, so motion correction has to be limited to subject‐level motion correction methods.

As recommended by several previous papers, we think that there are merits to having different preprocessing pipelines for groups with different motion profiles as well as when performing different analyses, as no single preprocessing procedure is ideal for all cases. Some factors which need to be considered for the acquisition and processing of rs‐fMRI data include the repetition time TR, use of slice time correction, the imaging sequence to capture the BOLD signal, head motion criteria to include a subject fMRI data in the study, the motion profile of the sample and the population to be studied, the model complexity for modeling head motion, the use of GSR, the threshold used to decide motion corrupted volumes, the number of time points left after motion censoring required for the stable estimation of RSFC metrics, the use of subject‐level motion correction versus group‐level motion correction, and the use of group‐level motion correction, if the variable of interest is correlated with head motion. The second important factor to consider is the objective and analysis of the study. As we have discussed earlier, motion censoring effectively precludes many types of analyses such as the ones that use hemodynamic deconvolution. Although interpolation has been suggested to reconstruct the removed time points, the fit could be unreliable as the neighboring points of a motion corrupted time point may also be corrupted by motion since multiple time points are affected by head motion. Another example relates to the use of group‐level motion correction in analyses involving clinical populations, especially in hyperkinetic populations where disease status is associated with head motion. Group‐level correction of head motion might remove some of the disease‐related variance. Therefore, a proper choice of the processing pipeline based on the motion profile and the planned analyses can reduce motion artifacts while still achieving study objectives.

The relationship between head motion and brain connectivity is a bi‐directional relationship, that is, differences in brain connectivity could be associated with head motion in the scanner (Zeng et al., 2014) just as head motion could cause artifactual changes in connectivity. Some have hypothesized that this might suggest that reduced connectivity in regions corresponding to the DMN might predict how still the person can stay in the scanner (Zeng et al., 2014). These neural correlates of motion can cause functional connectivity changes that represent genuine variations of neural activity in certain regions, which can be mistaken for a motion artifact. Other areas such as the regions in the visual cortex have also been speculated to be a neural correlate of head motion (Pujol et al., 2014). Different clinical populations exhibit characteristic spatio‐temporal motion patterns that can be associated with distinct motion artifacts for various pathological conditions (Spisák et al., 2014) thus really complicating the distinction between disease changes in connectivity and motion artifacts and limiting the use of functional connectivity as effective disease biomarkers (Spisák et al., 2014). Given this scenario, it is all the more advantageous to prospectively correct for motion so that the resulting data undergoes as little retrospective correction as possible, so that the component of motion‐related changes that may represent system‐specific neural activity are preserved.

The observed BOLD signal is a convolution of the latent neural fluctuations with the hemodynamic response function (HRF, Handwerker, Ollinger, & D'Esposito, 2004; Wu et al., 2013). Resting state BOLD data could be deconvolved (Wu et al., 2013) to remove the spatial heterogeneity in the latency of the HRF. The fidelity of deconvolution can be affected not only by motion censoring (scrubbing), but also when motion artifacts are present in the data. Therefore, sufficient subject level motion correction must be performed at the individual level, and it should be ensured that the data are free from motion artifacts before deconvolution is performed to infer the latent neuronal activity.

### 4.3 Other motion correction methods

Many advances in retrospective motion correction methods, which involve slight modifications in the traditional preprocessing pipeline, have been reported to be beneficial in ameliorating motion artifacts. These methods can be used in combination with PACE for more effective reduction of motion artifacts. They include the usage of time series based or wavelet‐based despiking (Patel et al., 2014), using aCompCor (anatomical ComCor, Muschelli et al., 2014), or nuisance signal regression instead of mean CSF and WM signals, using edge voxel information rather than traditional motion parameters (Patriat, Molloy, & Birn, 2015). ANATICOR, which uses local WM regressors coupled with despiking (Jo et al., 2013), and ensures uniform smoothing in the entire data to further reduce the effects of interindividual differences in head motion (Scheinost, Papademetris, & Constable, 2014). The voxel‐wise estimates of head motion are derived from volume‐based realignment parameters and their accuracy is limited by the accuracy of the estimation of the volumetric realignment parameters. Therefore, slice wise parameter measures might give a better estimate of the actual voxel‐wise motion for every voxel in the brain. As rapid head movements between TRs can have a differential effect on different slices in a single volume and cannot be adequately modeled by volume‐based realignment parameters, use of these slice‐wise estimates may aid in the calculation of voxel‐wise displacements and correction of motion‐induced signal changes (Beall & Lowe, 2014). While comparing motion‐prone clinical populations with healthy controls at the group level, the use of regional displacement interaction, which would encapsulate motion information in the voxel‐wise metrics rather than use a summary motion statistic could further correct for motion artifacts and preserving neuronal effects (Spisák et al., 2014).

Other methods for motion correction involve the use of multi‐echo sequences which require slight modifications of the scanning sequence compared to typical EPI scans. Regressing a voxel‐wise short TE scan from BOLD contrast could be used to remove motion and physiological noise from the BOLD signal (Bright & Murphy, 2013) or use more integrated approaches combining multi‐echo scans, ICA and separation of BOLD related components from non‐BOLD components (Kundu et al., 2013).

An evaluation of the acquisition strategies with a combination of preprocessing procedures will probably result in the best way to reduce motion artifacts in the BOLD data. It is also important to note that the success of better imaging sequences should not preclude the search for better retrospective motion correction methods for the data already collected and in cases, where prospective motion correction may not be possible. Since there are changes in the BOLD signal due to motion, it could potentially affect not just static functional connectivity measures, but also other measures such as DFC estimates, EC and multivariate pattern analyses results. A thorough investigation is required as to how the changes in signal intensity propagate into higher analyses to cause specific and structured artifacts.

### 4.4 Limitations

The number of subjects we have used for this study (*N* = 47) is reasonable for typical fMRI studies, but small compared to other reports which have evaluated retrospective strategies using large databases (*N* > 100). Due to the nature and effect sizes of motion artifacts, sample size can have a bearing on the results. Therefore, our results should be confirmed with a larger sample. Also, phenotypic factors such as age can have a bearing on motion artifacts. Our sample was homogeneous in this respect (20 male/27 females, age 25.1 ± 5 years) and hence did not sample the entire spectrum observed in the general population. Since we did not use external motion tracking devices to quantify head motion, the accuracy and reliability of image‐based motion metrics used for prospective and retrospective correction of head motion could not be independently validated. Since PACE is a prospective motion correction method, we might not know the actual head movement of the subject, but only the residual motion of the subject on the scanner coordinates. Another limitation of the PACE sequence owes to the fact that the types of head‐motion corrected by prospective image‐based motion correction is limited. PACE is effective for slow‐drifting motion and may not be able to correct for short jerky movements of the head. Due to the nature of the sequence, the scanner may take several TRs to adjust the scanner coordinates after the head movement has subsided. In this paper, we only show the results for PACE‐corrected data because even if we had non‐PACE data from the same subjects, they would not be comparable with PACE data as the head motion, though correlated is not reproducible across runs within subjects. Therefore, it is impossible to directly compare data with and without PACE correction in a time‐locked manner. Further studies should consider direct comparisons of prospective motion correction strategies with traditional EPI protocols and retrospective methods.

## CONFLICT OF INTEREST

The authors declare that there is no conflict of interest.

## Data Availability

The data that support the findings of this study are openly available in NITRC at https://www.nitrc.org/projects/epi_pace_rest/.
